# Burden and clinical profile of genetic eye diseases in children in Nigeria: a descriptive cross-sectional study

**DOI:** 10.11604/pamj.2023.45.150.40668

**Published:** 2023-08-04

**Authors:** Henrietta Ifechukwude Monye, Olusola Oluyinka Olawoye, Mary Ogbenyi Ugalahi, Tunji Sunday Oluleye, Adeyinka Olusola Ashaye

**Affiliations:** 1 University College Hospital, Ibadan, Nigeria,; 2 Eleta Eye Institute, Ibadan, Nigeria,; 3 Department of Ophthalmology, College of Medicine, University of Ibadan, Ibadan, Nigeria,; 4 Department of Ophthalmology, University College Hospital, Ibadan, Nigeria

**Keywords:** Genetic eye diseases, pedigree, ophthalmic genetics, children

## Abstract

**Introduction:**

ophthalmic genetics is rapidly evolving globally but is still nascent in much of sub-Saharan Africa, with gaps in knowledge about the burden in the region. This study evaluated the burden and manifestations of genetic eye diseases in children in Ibadan, Nigeria.

**Methods:**

this was a hospital-based cross-sectional study in which new and follow-up paediatric eye clinic patients were recruited consecutively at the University College Hospital, Ibadan. Children with genetic eye diseases had comprehensive ocular and systemic examinations, and their pedigrees were charted to determine the probable modes of inheritance. The main outcome variables were the proportion of study participants with genetic eye diseases, the probable modes of inheritance, and the clinical diagnoses. Summary statistics were performed using means and standard deviations for numerical variables and proportions for categorical variables.

**Results:**

fifty-two (12%) of 444 children had genetic eye diseases, and their mean (SD) age was 88.8 ± 50.4 months. Thirteen different phenotypic diagnoses were made following the evaluation of the 52 children, including primary congenital glaucoma (13, 25%) and familial non-syndromic cataracts (8, 15%). The probable modes of inheritance were derived from the pedigree charts, and 30 (58%) conditions were presumed to be sporadic.

**Conclusion:**

this study demonstrated a significant burden and a wide range of paediatric genetic eye diseases in this tertiary referral centre in Nigeria. This information provides invaluable evidence for planning ophthalmic genetic services.

## Introduction

There has been a gradual shift in the aetiology of childhood blindness in developing countries such as Nigeria from communicable diseases such as measles with corneal scarring [1,2], to non-communicable diseases. These non-communicable diseases include inherited eye diseases like congenital cataracts, childhood glaucomas, inherited retinal diseases, and retinoblastoma [3,4]. For instance, congenital cataracts are the commonest cause of childhood blindness in Nigeria [3], and about 50% of congenital cataracts may have a genetic basis [5].

The molecular diagnosis of genetic eye diseases holds much promise in ophthalmology practice through accurate diagnosis and understanding of recurrence risk and prognosis [6]. Consequently, this enhances the quality of genetic counselling and provides invaluable evidence for possible treatment, enrollment in clinical trials and further research [6]. Furthermore, it may pave the way for novel strategies aimed at pre-symptomatic detection or gene-directed treatment.

There has been remarkable advancement globally in the field of ophthalmic genetics in terms of clinical and molecular diagnoses, genetic counselling, clinical trials, and gene therapies for inherited diseases such as Leber congenital amaurosis, achromatopsia, choroideraemia, X-linked retinitis pigmentosa, and Stargardt disease [7,8]. However, it is still quite nascent in low- and middle-income countries, including Nigeria, as regards research, resources, and service provision. Generating evidence of the need for a service is a vital step in planning for health services and utilising this evidence is a major determinant of the success of the services [9]. There is currently a dearth of information on the burden of genetic eye diseases and their patterns of clinical presentation in children in Nigeria. It is important to generate evidence for paediatric genetic eye diseases to enhance cost-effective and evidence-based decisions for policy and planning.

Although various studies in Nigeria have examined the burden of congenital ocular anomalies, these studies were largely retrospective and made no distinction between anomalies of genetic origin and those of other aetiologies [10-18]. In the early 90s, Gilbert *et al*. [19] assessed the aetiology of hereditary eye diseases in developing countries, including three West African countries (Ghana, Togo and Benin) and reported a prevalence of 11-32%. However, the study was carried out on children in schools for the blind. Similarly, Olowoyeye *et al*. [4] examined the aetiology of blindness and visual impairment in two schools for the blind and visually impaired in Lagos, Nigeria, and attributed a genetic aetiology to 11.2% (13 of 116) of the cases. The aim of this study was to specifically evaluate the burden, clinical profiles, and modes of inheritance of genetic eye diseases among a paediatric population at a tertiary eye hospital setting in Nigeria in order to provide data for policy and planning.

## Methods

**Study design and setting:** the study was a hospital-based, cross-sectional, descriptive study carried out at the eye clinic of the University College Hospital (UCH), Ibadan, a major referral tertiary centre in Southwest Nigeria. Participants were recruited from April to July 2021.

**Participants:** the study population comprised new and follow-up patients below 18 years of age who presented to the eye clinic, UCH, Ibadan, during the study period. Participants were recruited consecutively using a total sampling approach till the study sample size was achieved. Patients with genetic eye diseases according to the study definition were further evaluated. Their family and clinical histories were obtained and their pedigrees were charted up to at least three generations to determine their probable inheritance pattern. Subsequently, they underwent further comprehensive ocular and systemic examinations as appropriate. The ocular examination involved anterior segment slit-lamp examination (Haag-Streit), intraocular pressure measurement (Goldman Applanation Tonometry), dilated fundoscopy with 78D lens, and/or binocular indirect ophthalmoscope as applicable. Appropriate systemic examinations were also carried out. A clinical (phenotypic) diagnosis was then made based on the history and clinical evaluation.

**Variables:** the main outcome variables of interest were the proportion of study participants with genetic eye diseases, the modes of inheritance of the genetic eye diseases and the clinical diagnoses.

**Study definitions and diagnostic criteria:** the study definition of genetic eye disease was restricted to chromosomal and single gene (Mendelian) but not complex or multifactorial diseases since routine genetic testing for the latter is not currently recommended till benefits can be demonstrated for specific types of therapy and surveillance [20]. Chromosome disorders were defined as diseases due to an excess or a deficiency of the genes located on entire chromosomes or chromosome segments [21]. Mendelian or single gene disorders were defined as diseases caused by pathogenic mutations in individual genes which may be present in one or both pairs of chromosomes and are inherited in a Mendelian pattern (autosomal recessive or dominant, or x-linked) [21].

Inheritance patterns were described as autosomal dominant or autosomal recessive. Genetic diseases that are not known to be inherited, or which were thought to be due to de novo mutations were categorised as "sporadic". The basic definitions used for these inheritance patterns are as follows [21]:

***Autosomal dominant:*** the phenotype occurs in every generation and is transmitted from either parent to offspring of either sex.

***Autosomal recessive:*** the phenotype may occur in siblings of the proband but not in parents, offspring or other relations, affects both sexes equally, and parents may be consanguineous.

***Sporadic:*** the disease is not the result of the inheritance of a disease-causing allele but is often due to a new germline or a somatic mutation.

The study definition for childhood cataracts of genetic origin was based on the presence of a definite family history, in the absence of any history or finding suggestive of any other known aetiology. These criteria were necessary to avoid misclassification because of the complexity of accurately diagnosing the underlying aetiology of congenital cataracts in the absence of a molecular diagnosis [22,23], which was beyond the scope of this study.

**Data source and measurements:** data were collected with a pre-designed proforma. The proportion with genetic eye diseases was calculated as a proportion of the total number of participants recruited. The modes of inheritance for each participant with a genetic eye disease was determined from the participant's pedigree chart which was constructed up to at least three generations. The clinical diagnoses were determined following careful clinical examination.

**Bias:** care was taken to ensure that participants were recruited only once in the course of the study.

**Study size:** this was based on the proportion of suspected ocular genetic diseases among children in a general paediatric ophthalmology clinic catering for an ethnically diverse inner-city area in the United Kingdom of 13% [24]. The sample size estimation formula for cross sectional studies with categorical variables was employed as follows:


$$n=\frac{Z^{2}P\left(1-P\right)}{d^{2}}$$

where n was the estimated minimum sample size, Z was the standard normal variate at 95% confidence interval (1.96) and 80% power (0.84), proportion of children with genetic eye diseases in a clinical setting from a previous study (13%) [24], and d was the level of precision (5%). The calculated minimum sample size was 355. The final study sample size was increased by 20% to 444 to account for non-response.

**Quantitative variables and statistical methods:** data management was done using the IBM Statistical Package for Social Sciences (SPSS) version 20.0 (IBM Corp., Armonk, NY). Summary statistics were performed using means and standard deviations for numerical variables and proportions for categorical variables. The proportion of participants who had genetic eye diseases was calculated from the total number of participants recruited for the study.

**Ethical considerations:** ethical approval was obtained from the Ethics Review Committee of the University College Hospital, Ibadan (UI/EC/20/0368). Informed consent was obtained from the parents of participants, and assent from participants > seven years old if they were mentally capable of granting it.

## Results

**Participants and demographic data:** a total of 444 children were recruited over a period of four months. Their ages ranged from one to 204 months with a mean (SD) of 88.8 ± 50.4 months, and 244 (55%) were males.

### Outcome data

***Proportion of children with genetic eye diseases:*** the proportion of children that were presumed to have genetic eye diseases was 12% (n=52, 95% confidence interval: 8.8% - 14.6%). Their ages ranged from five to 168 months with a mean (SD) of 74.4 (49.2) months. There was no history of consanguinity in any of the parents.

***Clinical diagnoses and modes of inheritance:*** overall, 13 different phenotypic diagnoses were made following the evaluation of the 52 children with presumed genetic eye diseases. Their modes of inheritance were presumed to be sporadic in 30 (58%), autosomal dominant in 19 (36%), and autosomal recessive in 3 (6%) children, respectively, based on their clinical histories and pedigrees. The demographic details, clinical features and probable modes of inheritance of the children with genetic eye diseases are shown in Table 1. The most common conditions encountered were primary congenital glaucoma (15, 25%), familial non-syndromic cataracts (8, 15%) and aniridia (6, 11%). Some of the pedigrees of interest include those for familial non-syndromic cataracts, aniridia, retinoblastoma, Marfan syndrome, and familial retinal detachment.

**Table 1:** summary of the demographic and clinical features of the participants

S/N	Diagnosis	Frequency (%)	Probable mode of inheritance	Family history	Systemic association	Sex	Age range (months)	Laterality
1	Primary congenital glaucoma	15 (25)	Sporadic (all)	No (all)	No (all)	Males (9) Females (3)	3 -120	Bilateral (all)
2	Familial non-syndromic cataract	8 (15)	Autosomal dominant (all)	Yes (all)	No (all)	Male (1) Females (7)	22 - 108	Bilateral (all)
3	Aniridia (isolated)	6 (11)	Autosomal dominant (all)	Yes (all)	No (all)	Males (3) Females (3)	13 - 144	Bilateral (all)
4	Sturge Weber syndrome	4 (8)	Sporadic (all)	No (all)	Yes (all)	All males	24 - 30	Unilateral (all)
5	Retinitis pigmentosa (non-syndromic)	4 (8)	Sporadic (all)	No (all)	No (all)	Male (1) Females (3)	108 - 144	Bilateral (all)
6	Retinoblastoma (intraocular)	4 (8)	Sporadic (3) Autosomal dominant (1)	Yes (3) No (1)	No (all)	Male (1) Females (3)	11 - 32	Bilateral (1) Unilateral (3)
7	Oculocutaneous albinism	3 (6)	Autosomal recessive (all)	Yes (all)	No (all)	Males (2) Female (1)	15 - 60	Bilateral (all)
8	Marfan syndrome	3 (6)	Autosomal dominant (2) Sporadic (1)	Yes (2) No (1)	Yes (all)	Males (2) Female (1)	156 - 168	Bilateral (all)
9	Down syndrome	2 (4)	Sporadic (all)	No (all)	Yes (all)	Male (1) Female (1)	108 - 156	Bilateral (all)
10	Juvenile open angle glaucoma	2 (4)	Autosomal dominant (all)	Yes (all)	No (all)	Females (2)	120 - 156	Bilateral
11	Bardet Biedl syndrome	1 (2)	Sporadic	No	Yes	Male (1)	108	Bilateral
12	Neurofibromatosis (NF-1)	1 (2)	Sporadic	No	Yes	Male (1)	120	Bilateral
13	Familial retinal detachment	1 (2)	Autosomal dominant	Yes	No	Male (1)	108	Bilateral

**Figure 1:**
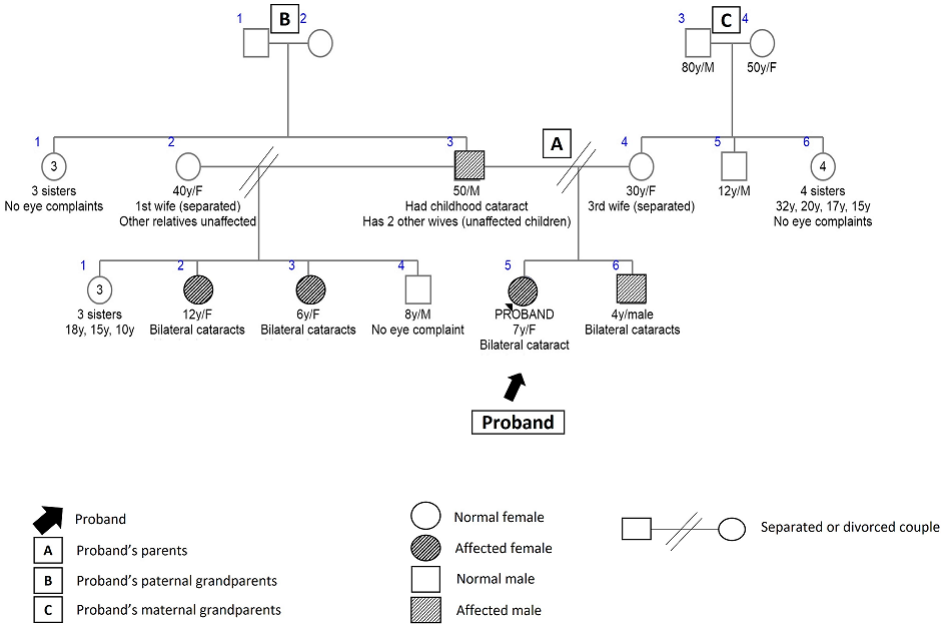
pedigree for familial non-syndromic cataract

The children with familial non-syndromic cataracts (8,15%) included three sibling trios - a pair of female twins aged 22 months and their four-year-old female older sibling; two stepsisters aged six and seven years; and three other unrelated children. They were all presumed to have autosomal dominant mode of inheritance because of a positive family history of the condition in a parent and other family members in multiple generations. The pedigree for the step sisters is shown in Figure 1. They had a positive history of bilateral childhood cataracts in their father, the twelve-year-old female sibling of one of them, and the four-year-old male sibling of the other.

**Figure 2:**
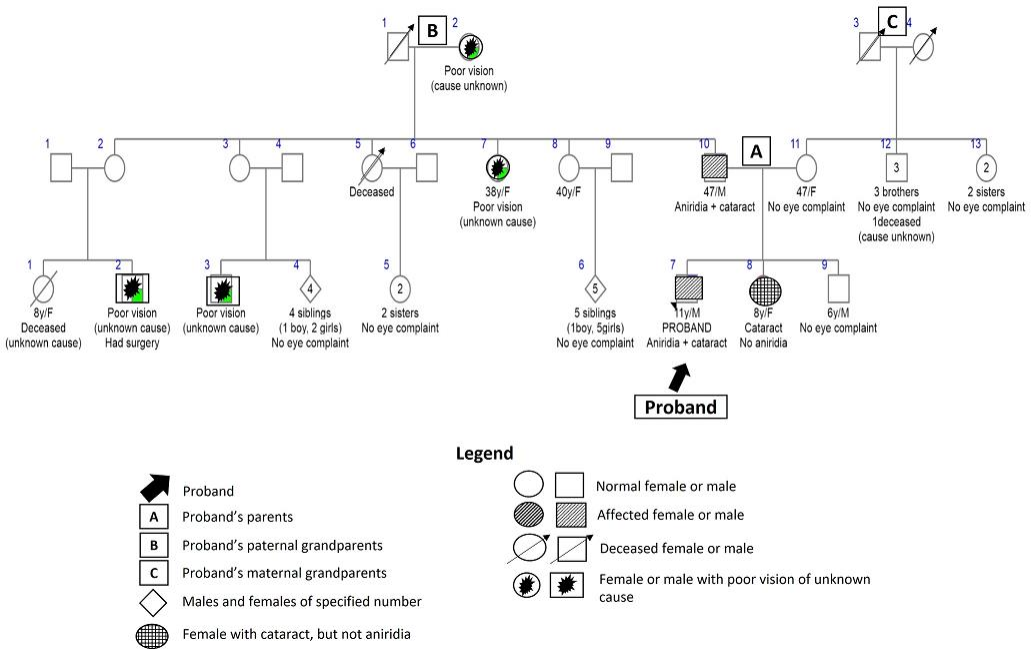
pedigree for aniridia

Six (11%) children had isolated bilateral aniridia with autosomal dominant pattern of inheritance based on their pedigrees and lack of systemic associations. They included a sibling trio comprising a six-year-old female, an eight-year-old male, and a 12-year-old female; a sibling pair comprising a five-year-old female and a 13-month-old male; and an 11-year-old male. The 11-year-old male child had bilateral aniridia and cataracts (pseudophakic), had an eight-year-old sister with bilateral developmental cataracts but no aniridia, a history of bilateral aniridia and cataract in his father, and poor vision in an aunt and two male cousins from his paternal side (Figure 2).

**Figure 3:**
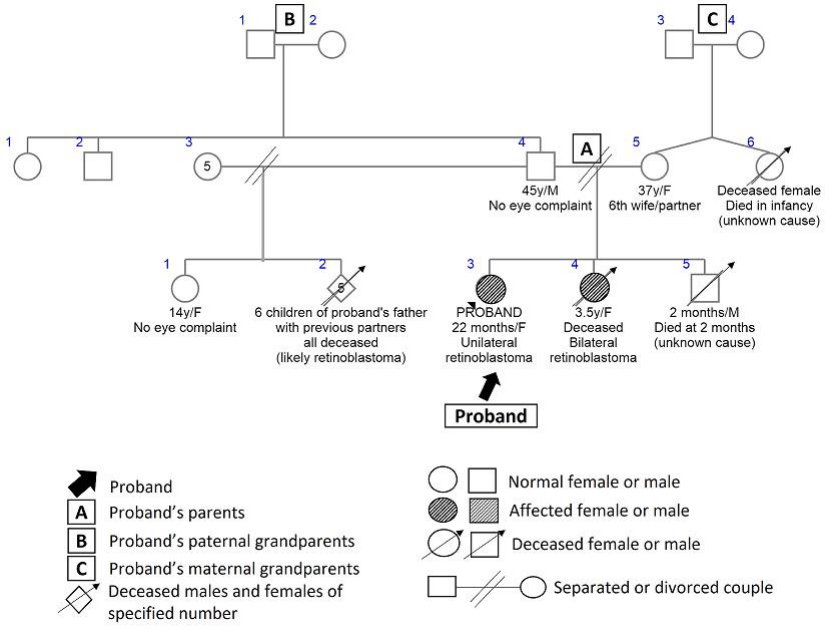
pedigree for retinoblastoma

Four (8%) children had intraocular retinoblastoma - one bilateral and three unilateral. Three of them were considered to have sporadic or new mutations (two of the three with unilateral disease and the child with bilateral disease). The remaining child with unilateral disease was presumed to have an autosomal dominant pattern of inheritance because of a positive history of bilateral disease in an elder sibling, and probable history of retinoblastoma in six deceased step siblings (father's children with previous partners (Figure 3). The father was presumed to be a silent carrier of the mutation.

**Figure 4:**
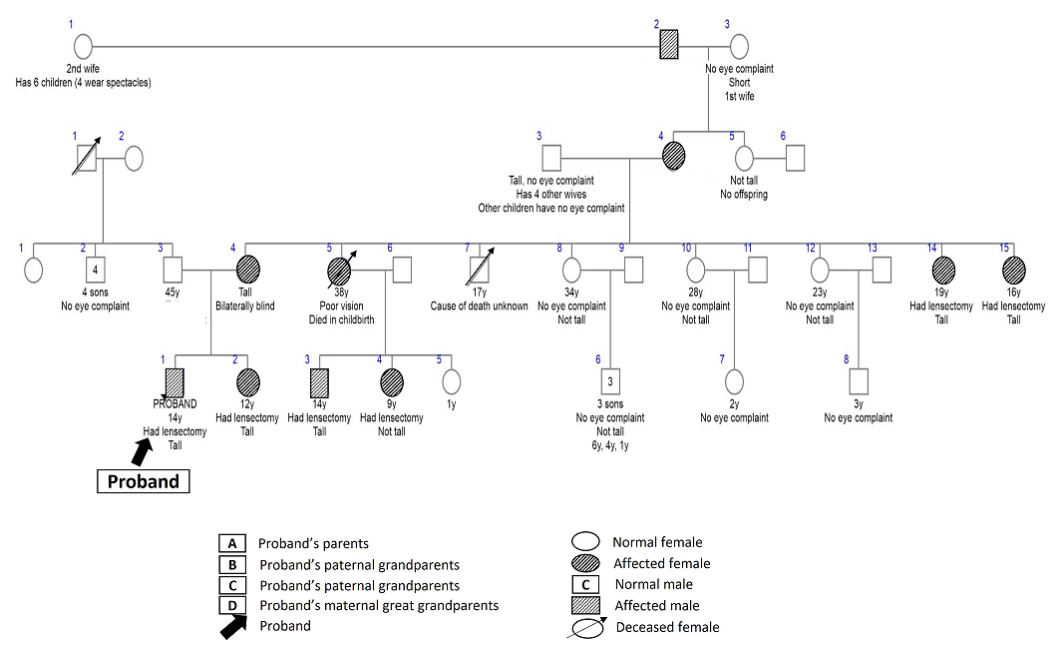
pedigree for Marfan syndrome

Three (6%) children had Marfan syndrome - a male and female sibling pair aged 14 and 13 years, respectively, who were presumed to have an autosomal dominant pattern of inheritance because of a strong family history in a parent and in multiple relatives (Figure 4). The third child was a 14-year-old male with no family history who was presumed to have a sporadic disease.

**Figure 5:**
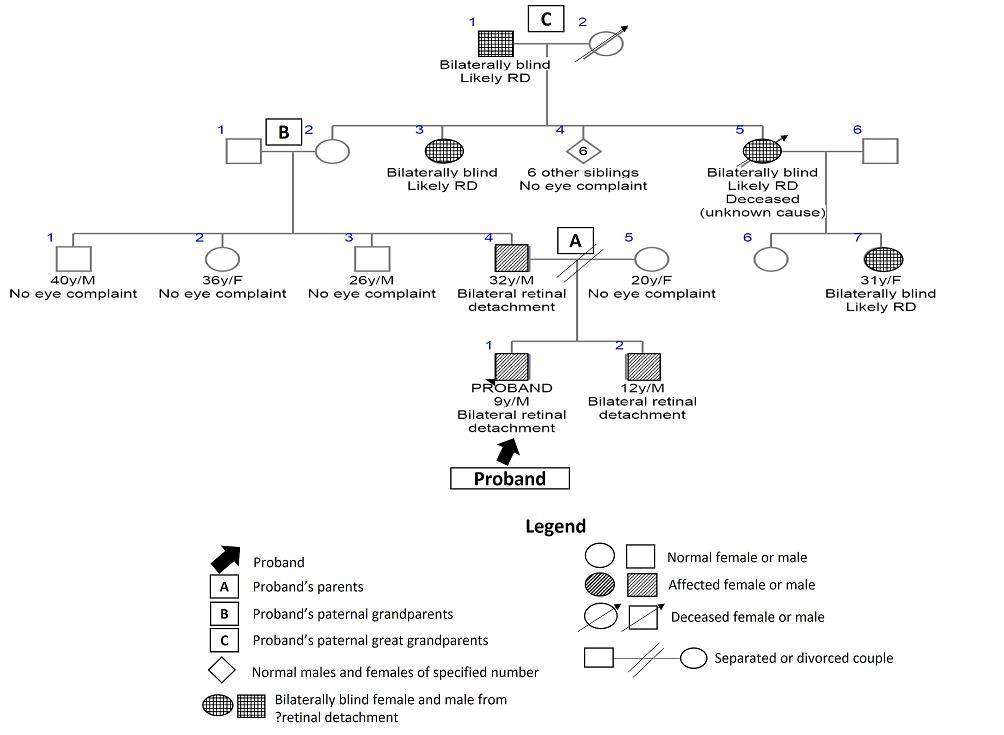
pedigree for familial retinal detachment

Furthermore, a nine-year-old male child with familial, non-syndromic retinal detachment was presumed to have an autosomal dominant pattern of inheritance (Figure 5). He was bilaterally blind from bilateral retinal detachment, and his 12-year-old brother also had bilateral retinal detachment. In addition, he had a positive family history of similar bilateral blindness at a young age in four generations of his paternal relations - great-grandfather, two grand-aunts, father, and aunt.

## Discussion

This study assessed the proportion, pedigrees, probable modes of inheritance and clinical profiles of children with genetic eye diseases at the University College Hospital, Ibadan. The proportion of children with genetic eye diseases was 12% (52), similar to the 13% reported by Reddy *et al*. [24] who also studied children with genetic eye diseases in a clinical setting - a general paediatric ophthalmology clinic in a multi-ethnic population in East London. Forty-four (13%) of their participants were Africans or black.

Pedigree charts served as a guide in this study to determine a probable pattern of inheritance for each patient with a genetic eye disease, and whether a systemic association was expected for conditions with multiple patterns of inheritance such as aniridia. The conditions were presumed to be sporadic in a majority (30, 58%) of the children, autosomal dominant in 19 (36%), and autosomal recessive in 3 (6%). This served as a basis for counselling the parents of these children on the possible risks of recurrence in future offspring as well as risks to other family members. Comprehensive pedigree charting is a vital component of the genetic evaluation process, and can save both time and money in reaching a diagnosis [25]. It also provides invaluable information that can guide management and reproductive choices especially in resource-limited settings like Nigeria where routine genetic testing is not available.

Furthermore, clinical evaluation, comprising comprehensive family history, pedigree charting, clinical examination, and investigation, is still a fundamental aspect and the first step of any genetic evaluation, even when sophisticated cytogenetic and molecular genetic tests are available. Clinical diagnosis is an important aspect of genetic evaluation as it can help guide the choice of genetic testing. Likewise, genetic test results should be interpreted in light of clinical findings [26]. In resource-constrained settings like Nigeria, presumptive clinical diagnosis and documentation may be particularly invaluable in cost-effectively directing the choice of appropriate genetic tests and patient enrollment for clinical trials and gene therapy when the opportunity arises.

The most common of the 13 forms of phenotypic diagnoses encountered in this study were primary congenital glaucoma (13, 25%) and non-syndromic familial cataracts (8, 15%). Reddy *et al*. [24] in London noted that Down's syndrome (7, 15%) and non-syndromic retinitis pigmentosa (4, 9%) were the commonest pathologies in their study. The differences noted in aetiology somewhat reflect the common global aetiological pattern in which neurological and retinal diseases are more prevalent in developed countries [27] while cataracts and glaucoma are more common in developing countries [28].

Olowoyeye *et al*. [4] and Gilbert *et al*. [19] reported on the genetic aetiology of blindness and visual impairment in schools for the blind and visually impaired in Lagos, Nigeria (2017), and in West Africa (Togo, Ghana, and Benin; 1990-1994) respectively. Hereditary cataracts occurred most frequently in the study by Olowoyeye *et al*. [4]. Mirroring the finding in this study in which familial non-syndromic cataract was the second most common diagnosis. Conversely, retinal dystrophies were the commonest hereditary aetiology in the study by Gilbert *et al*. [19]. The discrepancy in aetiology may be due to the enrollment pattern in blind schools at that time which may have been more accessible to children from the higher socioeconomic class.

Though consanguinity may be linked to an increased prevalence of genetic eye diseases as highlighted by Kemmanu *et al*. [29] in the Pevagada Eye Disease Study 2 in children in Southern India, there was no history of consanguinity among the parents of the children with genetic eye diseases in this study. A greater burden of genetic eye diseases, particularly autosomal recessive forms has been shown to be associated with consanguinity in different populations [30-32].

**Limitations:** a limitation of this study is a probable underestimation of the proportion of children with genetic eye diseases because of the strict criteria employed for the diagnosis of cataracts of genetic origin. Molecular diagnosis was beyond the scope of this study. Furthermore, being a hospital-based study, the proportion reported only accounts for the population of people who have access to, and have been able to seek health care. This may therefore represent only the tip of the iceberg of the burden of genetic eye diseases in the general population.

## Conclusion

This study showed that, indeed, there is a significant burden of paediatric genetic eye diseases in Nigeria. It included both new and follow-up patients and, therefore, represents the likely burden of genetic eye diseases in a paediatric ophthalmology eye clinic. This may be useful in terms of policy and planning for ophthalmic genetic services. Furthermore, though routine genetic testing for ocular diseases is not yet available in Nigeria, this study has demonstrated that careful pedigree charting and clinical evaluation can provide invaluable information that can aid clinical diagnosis and counselling. Finally, this study underscores the importance of incorporating genetic services into paediatric ophthalmic care in lower-income countries like Nigeria as this would ensure that more accurate diagnoses can be made in a timelier and cost-effective manner. It would also enable better counselling of parents of children with genetic eye diseases so that they can make informed management and reproductive choices going forward. We recommend the incorporation of routine pedigree charting in the evaluation of children suspected to have ocular genetic diseases, and ophthalmic genetics services as part of paediatric ophthalmology care in Nigeria to ensure a more holistic evaluation that would guide management and enhance outcomes in these children. A national genetic eye disease register will be vital in providing evidence of the burden in Nigeria for policy making and will ensure easy communication with patients in case of future opportunities for genetic testing, clinical trials, and other technological advancements. Finally, a large collaborative hospital-based study with molecular diagnosis across Nigeria may be necessary to provide more comprehensive evidence on the burden and manifestations of paediatric ocular genetic diseases in the country to aid policy and planning.

### What is known about this topic

- *Despite the recent advances in the field of ophthalmic genetics globally, much of sub-Saharan Africa is still underrepresented in this field of research;*
- *Little is known about the burden and manifestations of genetic eye diseases children in the region*.

### What this study adds

- *This study demonstrates a significant burden and variety of genetic eye diseases among children in a clinical setting in Nigeria;*
- *It provides evidence to aid planning of ophthalmic genetic services in similar settings*.
